# Efficacy of an AAV vector encoding a thermostable form of glucocerebrosidase in alleviating symptoms in a Gaucher disease mouse model

**DOI:** 10.1038/s41434-024-00476-8

**Published:** 2024-08-15

**Authors:** Ivan Milenkovic, Shani Blumenreich, Ariel Hochfelder, Aviya Azulay, Inbal E. Biton, Mirie Zerbib, Roni Oren, Michael Tsoory, Tammar Joseph, Sarel J. Fleishman, Anthony H. Futerman

**Affiliations:** 1 Department of Biomolecular Sciences, Weizmann Institute of Science, Rehovot, 7610001 Israel; 2 Department of Neurology, Medical University of Vienna, Vienna, Austria; 3 Department of Veterinary Resources, Weizmann Institute of Science, Rehovot, 7610001 Israel

**Keywords:** Metabolic disorders, Transfection

## Abstract

Almost all attempts to date at gene therapy approaches for monogenetic disease have used the amino acid sequences of the natural protein. In the current study, we use a designed, thermostable form of glucocerebrosidase (GCase), the enzyme defective in Gaucher disease (GD), to attempt to alleviate neurological symptoms in a GD mouse that models type 3 disease, i.e. the chronic neuronopathic juvenile subtype. Upon injection of an AAVrh10 (adeno-associated virus, serotype rh10) vector containing the designed GCase (dGCase) into the left lateral ventricle of *Gba*^*−/−*^*;Gba*^*tg*^ mice, a significant improvement in body weight and life-span was observed, compared to injection of the same mouse with the wild type enzyme (wtGCase). Moreover, a reduction in levels of glucosylceramide (GlcCer), and an increase in levels of GCase activity were seen in the right hemisphere of *Gba*^*−/−*^*;Gba*^*tg*^ mice, concomitantly with a significant improvement in motor function, reduction of neuroinflammation and a reduction in mRNA levels of various genes shown previously to be elevated in the brain of mouse models of neurological forms of GD. Together, these data pave the way for the possible use of modified proteins in gene therapy for lysosomal storage diseases and other monogenetic disorders.

## Introduction

Monogenetic diseases, such as the lysosomal storage diseases (LSDs), which are often caused by a single point mutation in one or other gene encoding a lysosomal protein [1], are ripe for genetic therapies [2]. Of the LSDs, Gaucher disease (GD) has received particular attention over the past 15 years or so due to the genetic association between mutations in *GBA*, which encodes the defective lysosomal enzyme in GD, acid-beta-glucosidase (GCase), and Parkinson’s disease (PD) [3], although little data is available to permit delineation of the cellular and biochemical basis of this association [4]. GD is typically divided into three clinical subtypes, with the juvenile neurological form (type 3) the target of novel forms of therapeutic intervention, such as substrate reduction therapy which partially inhibits synthesis of the accumulating substrate [5].

Gene therapy has also been attempted in the neurological forms of GD [6], including the other major neurological subtype, type 2 [7]. In all studies to date, gene therapy vectors encode the wild type (wt) sequence of recombinant GCase to attempt to reverse type 2 or 3 GD symptoms, with approaches optimized to allow access of the gene vector to the brain. Recently we designed a thermostable and highly expressible form of GCase [8], using the stability-design algorithm, PROSS, which combines atomistic Rosetta design calculations and phylogenetic sequence analysis to design stable variants [9, 10]. The designed protein, dGCase, with 55 mutations relative to the human GCase (wtGCase), had higher enzymatic activity than the clinically-used human GCase when incorporated into an AAV vector, resulting in longer-lasting activity and a larger decrease in the accumulation of lipid substrates, i.e. glucosylceramide (GlcCer), compared to wtGCase, in cultured *GBA*^−/−^ cells [8].

Using *Gba*^*−/−*^*;Gba*^*tg*^ mice, a model of type 3 GD developed in our laboratory [11], we now inject wtGCase or dGCase under the control of a ubiquitous CAG promoter in AAVrh10 capsids, into the left lateral ventricle and examine whether this vector can reverse the development of disease in the mice. Remarkably, *Gba*^*−/−*^*;Gba*^*tg*^ mice injected with AAV-dGCase lived much longer than mice injected with AAV-wtGCase, concomitant with better performance on a rotarod and in beam walk tests, reduction of brain inflammation as seen by MRI and a decrease in levels of inflammatory biochemical markers in the brain. Our results provide an encouraging indication that proteins enhanced using modern design algorithms could be used in other LSDs, particularly in the brain which has a minimal risk of an adversive immunological response, and perhaps in more common neurological diseases which show a genetic association to an LSD [12].

## Methods

### Animals

*Gba*^*+/+*^*;Gba*^*tg*^ and *Gba*^*−/−*^*;Gba*^*tg*^ mice, which contain a *Gba* transgene regulated by doxycycline, were generated and maintained as described [11], with two modifications (to enhance breeding efficiency) inasmuch as doxycycline was added to the drinking water of parental cages, rather than at six weeks after birth, and at a lower concentration (10^−7^ mg/ml). This affected the survival time of the mice, which now lived for 110–120 days after birth, with symptoms beginning to appear by ~60 days of age. Following breeding and stereotaxic injections (described below), four groups were used: *Gba*^*+/+*^*;Gba*^*tg*^ injected with PBS, *n* = 10; *Gba*^*−/−*^*;Gba*^*tg*^ injected with PBS, *n* = 10; *Gba*^*−/−*^*;Gba*^*tg*^ injected with AAV-wtGCase, *n* = 8; *Gba*^*−/−*^*;Gba*^*tg*^ injected with AAV-dGCase, *n* = 12. Group size was determined using the ‘pwr’ package in R [one-way ANOVA (analysis of variance) test for a general statistical power of 0.7, medium effect size of 0.5 and statistical significance level of 0.05], justifying a sample size of 10 mice per group. Procedures involving animals were reviewed and approved by the Institutional Animal Care and Use Committee of the Weizmann Institute of Science (IACUC application number 05310622-2), following guidelines issued by the Association for Assessment and Accreditation of Laboratory Animal Care. No randomization or blinding were performed.

### Genotyping

Male and female *Gba*^*+/+*^*;Gba*^*tg*^ and *Gba*^*−/−*^*;Gba*^*tg*^ mice were genotyped at 20–25 days of age, about a week prior to weaning. DNA was extracted [13] and PCR performed in a QuantStudio™ 5 (Applied Biosystems, Singapore); initial denaturation 95 °C, 3 min; 10 touchdown cycles with 15 s of denaturation at 95 °C, 15 s of annealing from 65 °C to 60 °C, and 10 s of extension at 68 °C; 28 cycles with 15 s of denaturation at 95 °C, 15 s of annealing at 60 °C, and 10 s of extension at 72 °C; and a final stage at 60 °C to 98 °C at a ramp mode set to 0.025 °C/sec (see Supplementary Table 1 for the primers used). Mice which survived until ~120 days of age were genotyped again for validation.

### AAV vectors

Vectors were generated at the translational vector core of the University Hospital of Nantes by packaging AAV2-based recombinant genomes containing DNA sequences encoding wtGCase or dGCase, under the control of a ubiquitous CAG promoter, into AAVrh10 capsids using helper virus-free transfection of HEK293 cells [8]. The vectors were purified using an optimized CsCl gradient-based purification protocol [14]. Viral protein purity and identity were verified by SDS-PAGE and silver staining, and vector titers quantified by qPCR with primers targeting the flanking sequence of ITR2.

### Stereotaxic injections

The method for stereotaxic injections was modified from ref. [15]. 35–38-day-old mice were injected intraventricularly into the left ventricle with 4 µl of AAV-wtGCase or AAV-dGCase [both at 1.1 × 10^13^ viral genome(vg)/ml] at a rate of 0.3 µl/min using a Hamilton syringe (10 µl with 26 G straight Hamilton needle, 22 mm PT3) to coordinates relative to the Bregma anterior-posterior (0.57 mm), medial-lateral (1.17 mm), dorsal-ventral (2.04 mm). Mice received painkillers for 2 days post-surgery (Carprofen^®^) and were monitored daily for distress, pain and discomfort. Mice were weighed and examined every day. Mice were humanely sacrificed if motor symptoms deteriorated, if they showed any other signs of distress or discomfort, or if they lost 20% of their body weight.

### Lipidomics

Homogenates of brain tissue were prepared as described [11]. Cell pellets were lysed in ultra-pure water containing a protease inhibitor cocktail (Sigma; 1:100) and homogenized using a GentleMACS dissociator (Miltenyi Biotec). Protein was measured by the BCA method prior to lyophilization. Quantitative analysis of GlcCer in lyophilates (600 µg protein/ml) was performed by liquid chromatography-tandem mass spectrometry [16].

### GCase activity

Enzymatic activity was determined using a fluorescently labeled substrate of GCase, C6-NBD-GlcCer [17, 18]. The assay was performed using homogenates (10 µg of protein) from brain tissue in a final volume of 20 µl McIlvaine buffer, pH 4.2. The reaction was run at 37 °C for 60 min and terminated by addition of 1.5 ml of chloroform-methanol (1:2, v/v) prior to lipid extraction.

### Behavioral tests

Motor function assessment was carried out in the middle of the dark-active phase of the diurnal cycle in designated experimental rooms. In order to minimize stress, mice were kept in their home cages for 1 h in habituation locker-like cabinets. Mouse coordination and balance were assessed by RotaRod (San-Diego Instruments) and beam walk tests.

Rotarod sessions consisted of 5–6 trials with an inter-trial interval of 1 min (adapted from Ref. [19]). In each trial, mice were placed on the rod while it was motionless and the rod accelerated the rotation speed from 0 to 40 r.p.m. over 4 min. Mice latency to fall off the rod was recorded and a mean score was calculated for each mouse based on the 3 best performances from all trials.

In the beam walk test [20], mice were first trained to walk on a plank (50 cm long and 35 mm wide), suspended 30 cm above the working surface. Following this, mouse balance and coordination were assessed five times while walking on the plank without stops or turns. These runs were recorded using an overhead camera and manually analyzed for the number of steps for each paw as well as the number of slips. The percent of slips per step was calculated. If mice were dragging their bodies or grasping the plank on the side, the run was designated as 100% slip.

### Magnetic resonance imaging

MRI experiments were performed on a 9.4 Tesla BioSpec Magnet 94/20 USR system (Bruker, Germany) equipped with a gradient coil system capable of producing a pulse gradient of up to 40 gauss/cm in each of the three directions. All MR images were acquired with a quadrature mouse head surface coil and transmitter linear coil (Bruker). The MRI protocol included high resolution multi-slice T_2_ weighted images and T_2_ maps. The T_2_ weighted images were acquired using the Rapid Imaging with Refocused Echoes (RARE) imaging sequence with the following parameters: a repetition delay of 1800 ms, effective time echo of 25 ms, RARE factor of 8, matrix dimension of 180 × 180 and 8 averages, corresponding to an image acquisition time of 5 min 28 s. Nineteen continuous slices with a slice thickness of 0.50 mm were acquired with a field of view of 1.8 × 1.8 cm^2^.

T_2_ maps were acquired using the multi-slice spin-echo imaging sequence with the following parameters: a repetition delay of 3000 msec, 16 time echo increments (linearly from 10 to 160 msec), matrix dimension of 256 × 128 (interpolated to 256 × 256) and 2 averages, corresponding to an image acquisition time of 12 min 48 s. The T_2_ dataset consisted of 16 images per slice. Thirteen continuous slices with a slice thickness of 0.90 mm were acquired with a field of view of 2 × 2 cm^2^. To evaluate volumetric changes between mouse groups, the whole brain and cerellbelum were segmented using the high-resolution T_2_-weighted images using Bruker ParaVision 360 software.

A quantitative T_2_ map was produced from the multi-echo T_2_-weighted images. The multi-echo signal was fitted to a mono-exponential decay to extract the T_2_ value for each image pixel. Image analysis was performed using homemade scripts written in Matlab R2013B. The procedure of co-registration (inter-subject and intra-subject) was applied before the MRI dataset analysis. For optimal suitability to a mouse brain atlas (correction of head movement image artifacts), all images went through atlas registration: reslicing, realignment and smoothing, using SPM software (version 12, UCL, London, UK). Voxel-by-voxel one-way analysis of variance [ANOVA; (*p* < 0.05)] comparison of the mice groups was done by SPM software. An effect was considered significant at *p* < 0.05. One-way ANOVA corrected for multiple comparisons with the false discovery rate approach (*p* < 0.05 following correction).

### Real-time PCR

Total RNA was isolated from three brain areas using a RNeasy mini kit (Qiagen, Germany) according to manufacturer’s instructions. cDNA synthesis was performed using a High-Capacity cDNA Reverse Transcription kit (Applied Biosystems, USA). Quantitative PCR was performed using the Fast SYBR™ Green Master Mix (Applied Biosystems, USA) and ABI Prism 7000 Sequence Detection System (Applied Biosystems, USA) with cDNA with equivalent of 5 ng of total RNA. The primer concentration was 100 nM in a reaction volume of 10 µl. The thermal cycling parameters were as follows: step 1, 95 °C for 10 min; step 2, 95 °C for 15 s; step 3, 60 °C for 30 s; step 4, 68 °C for 30 s. Step 2 was repeated for 40 cycles and was followed by a dissociation step. Primers are listed in Supplementary Table 2.

### Statistics

Statistical analysis was performed using R studio (version 2023.06.1 + 524). Data modeling was performed using “lme4” package (mixed-effect models) and “survival” package (Kaplan-Meier curve). Differences in mean values between groups were tested using ANOVA followed by Tukey’s Honest Significant Difference (HSD) post-hoc test. A mixed ANOVA model was applied in cases of 2 or more measured effects (using the ‘aov’ function). Assumptions of normality (Q-Q plot and Shapiro’s test) and equality of variances were tested (Levene’s test, package “car”). Graphs were generated using “ggplot2” package.

## Results

**Fig. 1:**
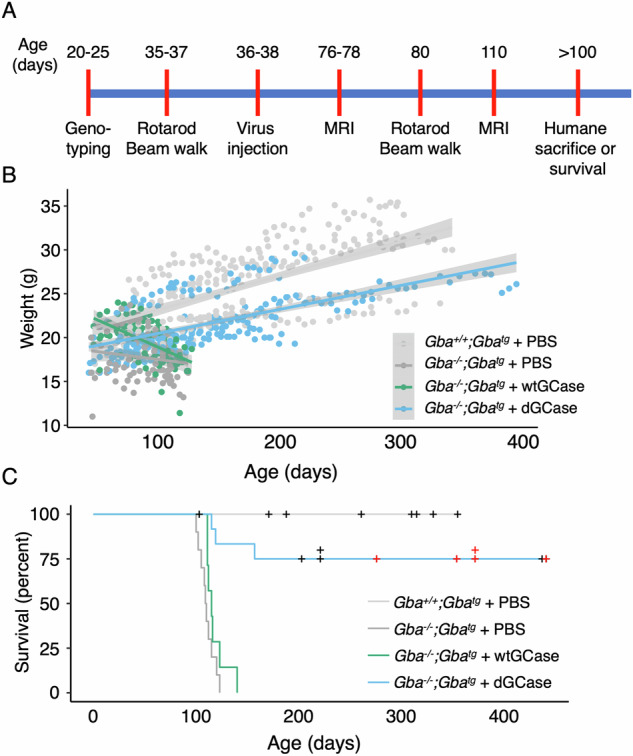
Effect of AAV-dGCase on mice survival. **A** Schematic of the study design. Mice were injected in the left lateral ventricle on days 36–38 with either PBS, AAV-wtGCase or AAV-dGCase [both at 1.1 ×1013 viral genome(vg)/ml]. *Gba*^*+/+*^*;Gba*^*tg*^ + PBS, *n* = 10; *Gba*^*−/−*^*;Gba*^*tg*^ + PBS, *n* = 10; *Gba*^*−/−*^*;Gba*^*tg*^ + AAV-wtGCase, *n* = 8; *Gba*^*−/−*^*;Gba*^*tg*^ + AAV-dGCase, *n* = 12. **B** Body-weight of all injected mice. The trend line represents linear regression for each group, grey areas are confidence intervals. Mice appear to divide into two groups, with males generally weighing more than females. A mixed-effect regression model demonstrated that the *Gba*^*−/−*^*;Gba*^*tg*^ + AAV-dGCase group gained 0.026 ± 0.001 g/day, similar to the *Gba*^*+/+*^*;Gba*^*tg*^ + PBS group (0.03 ± 0.001 g/day) whereas mice in the *Gba*^*−/−*^*;Gba*^*tg*^ + PBS group lost 0.03 ± 0.005 g/day and *Gba*^*−/−*^*;Gba*^*tg*^ + AAV-wtGCase mice lost 0.055 ± 0.005 g/day. **C** Kaplan-Meier curve showing mouse survival. Mice in the *Gba*^*−/−*^*;Gba*^*tg*^ + PBS and *Gba*^*−/−*^*;Gba*^*tg*^ + AAV-wtGCase groups were sacrificed when they met criteria indicated in the human endpoint of the experiment (i.e. if their motor function deteriorated or if they lost 20% of their maximum weight). Mice in the *Gba*^*+/+*^*;Gba*^*tg*^ + PBS group did not show disease symptoms and were sacrificed (+, *black*) at various times after PBS injection to collect biochemical material. Three *Gba*^*−/−*^*;Gba*^*tg*^ mice that were injected with AAV-dGCase were sacrificed at 115, 119 and 157 days, with the former and latter losing weight and the middle unable to eat chow due to a problem with its teeth; these are the not the same symptoms as displayed by *Gba*^*−/−*^*;Gba*^*tg*^ mice injected with PBS and may therefore not be disease-related. Four other mice (+, *black*) were sacrificed to obtain biochemical material and the remaining 5 mice were alive at the time of writing this manuscript (+, *red*). Note that data related to each individual mouse is documented in Supplementary Table 3.

In this study, *Gba*^*+/+*^*;Gba*^*tg*^ mice were injected in the left lateral ventricle with PBS and compared to *Gba*^*−/−*^*;Gba*^*tg*^ mice injected with PBS or with an AAVrh10 vector encoding either wtGCase or dGCase. Mice were injected 36–38 days after birth, immediately after they underwent baseline motor testing using rotarod and beam walk tests (Fig. 1A). Following injection, mice underwent MRI analyses and other behavioral tests, and were humanely sacrificied if their motor function deteriorated or if they lost 20% of their maximum weight. In some cases, mice were sacrificed in the absence of symptoms in order to obtain material for biochemical analyses (Fig. 1A). Information about the development of disease, behavioral tests and biochemical analyses for each individual mouse is documented in Supplementary Table 3.

*Gba*^*+/+*^*;Gba*^*tg*^ mice injected with PBS gained weight as expected for approximately one year before they were sacrificed (Fig. 1B, C). In contrast, *Gba*^*−/−*^*;Gba*^*tg*^ mice injected with PBS lost weight rapidly such that by ~110 days of age, they had lost close to 20% of their body weight (Fig. 1B, Supplementary Table 3) and were sacrificed (Fig. 1C). Likewise, *Gba*^*−/−*^*;Gba*^*tg*^ mice injected with AAV-wtGCase lost weight, had reduced mobility and displayed ruffled fur, similar to *Gba*^*−/−*^*;Gba*^*tg*^ mice injected with PBS, and were also sacrificed between 100 and 120 days. In contrast, *Gba*^*−/−*^*;Gba*^*tg*^ mice injected with AAV-dGCase continued to gain weight, although they weighed somewhat less than their *Gba*^*+/+*^*;Gba*^*tg*^ counterparts injected with PBS (Fig. 1B), with some mice living for > 450 days. Of the 12 *Gba*^*−/−*^*;Gba*^*tg*^ mice injected with AAV-dGCase (Supplementary Table 3), two mice were sacrificed at 115 and 119 days, respectively, with the former losing weight and the latter unable to eat chow due to a problem with its teeth. One other mouse was sacrificed at 157 days as it began to lose weight, but the remaining 9 *Gba*^*−/−*^*;Gba*^*tg*^ mice injected with AAV-dGCase lived for > 200 days, with 5 mice alive at 284, 362, 380, 380 and 449 days of age. Three *Gba*^*−/−*^*;Gba*^*tg*^ mice injected with AAV-dGCase were sacrificied at days 221 (two mice) and 203 (one mouse) to obtain brain tissue for biochemical studies. None of the latter 9 mice displayed overt signs of GD.

**Fig. 2:**
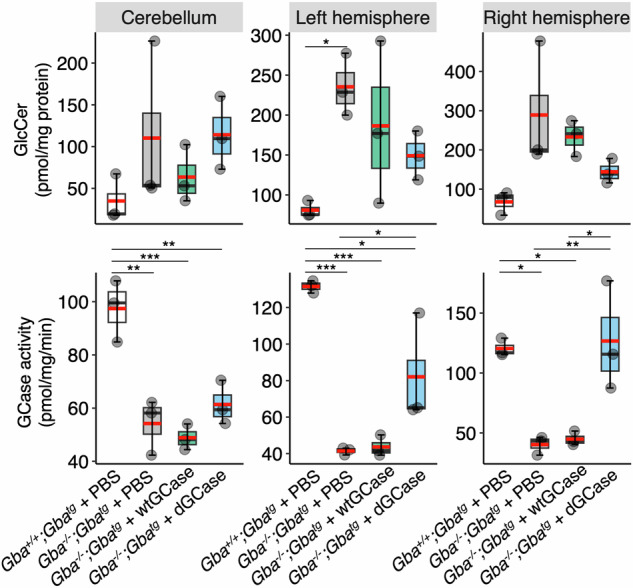
GCase activity and GlcCer levels. GlcCer levels (*upper* panel) and GCase activity (*lower* panel) was measured in three different brain areas. *Gba*^*+/+*^*;Gba*^*tg*^ + PBS, *n* = 3, 171 (two mice) and 261 days of age; *Gba*^*−/−*^*;Gba*^*tg*^ + PBS, *n* = 3, 105, 110 and 115 days of age; *Gba*^*−/−*^*;Gba*^*tg*^ + AAV-wtGCase, *n* = 3, 103, 112 and 140 days of age; *Gba*^*−/−*^*;Gba*^*tg*^ + AAV-dGCase, *n* = 3, 203, 221, 221 days of age. ANOVA was performed, followed by the Tukey HSD post-hoc test. ANOVA (F statistics) yielded the following results: GlcCer cerebellum, F_3,8_ = 1.25, *p* = 0.36; GlcCer left hemisphere, F_3,8_ = 3.93, *p* = 0.05; GlcCer right hemisphere, F_3,8_ = 3.72, *p* = 0.061; GCase activity in the cerebellum, F_3,8_ = 17.02, *p* < 0.001; GCase activity in the left hemisphere, F_3,8_ = 22.16, *p* < 0.001; GCase activity in the right hemisphere, F_3,8_ = 11.76, *p* < 0.01. Boxes represent lower quartile, median and upper quartile (*black*). The whiskers represent the minimum and maximum values, up to 1.5-times the interquartile range from the bottom or the top of the box to the furthest data point within that distance, thus excluding outliers. The mean is in *red*. *p ≤ 0.05; **p ≤ 0.01; ***p ≤ 0.001.

Lipidomics analysis of levels of GlcCer, one of the lipids that accumulates in GD [21], and examination of GCase activity, were performed on the four groups of mice. Since *Gba*^*−/−*^*;Gba*^*tg*^ mice injected with either PBS or AAV-wtGCase were sacrificed at ~100–120 days of age, and *Gba*^*+/+*^*;Gba*^*tg*^ mice injected with PBS or *Gba*^*−/−*^;*Gba*^*tg*^ mice injected with AAV-dGCase were sacrificed much later (170–260 days), the samples were not age-matched. Brains were divided into three regions, the cerebellum, the left hemisphere and the right hemisphere. In all of these regions, GlcCer levels were increased in *Gba*^*−/−*^*;Gba*^*tg*^ mice injected with PBS compared to *Gba*^*+/+*^*;Gba*^*tg*^ mice (Fig. 2), with a small reduction in *Gba*^*−/−*^*;Gba*^*tg*^ mice injected with AAV-wtGCase, although this did not reach control levels (i.e. *Gba*^*+/+*^*;Gba*^*tg*^ + PBS). A similar result was obtained in the cerebellum and left hemisphere of *Gba*^*−/−*^*;Gba*^*tg*^ mice injected with AAV-dGCase. In contrast, a greater reduction was seen in the right hemisphere in *Gba*^*−/−*^*;Gba*^*tg*^ mice injected with AAV-dGCase. The latter result is consistent with analysis of GCase activity, inasmuch as their GCase activity was closer to that of *Gba*^*+/+*^*;Gba*^*tg*^ mice injected with PBS, whereas *Gba*^*−/−*^*;Gba*^*tg*^ mice injected with AAV-wtGCase or with PBS showed a large reduction in GCase activity in all three brain regions (Fig. 2).

**Fig. 3:**
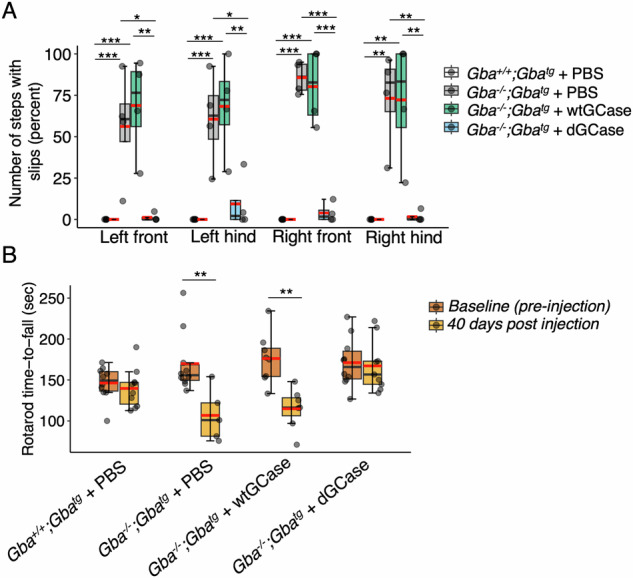
Beam walk and rotarod tests. **A** Percent of slips (relative to steps) for each of the four paws across groups, *Gba*^*+/+*^*;Gba*^*tg*^ + PBS, *n* = 5 (84, 87, 87, 87, 87 days of age); *Gba*^*−/−*^*;Gba*^*tg*^ + PBS, *n* = 4 (83, 83, 84 and 84 days of age); *Gba*^*−/−*^*;Gba*^*tg*^ + AAV-dGCase, *n* = 4 (83, 84, 85, 85 days of age); *Gba*^*−/−*^*;Gba*^*tg*^ + AAV-dGCase, *n* = 4 (83, 83, 85, 85 days of age). ANOVA was performed, followed by a Tukey HSD post-hoc test. ANOVA (F statistic), left front, F_3,13_ = 11.76, *p* < 0.001; left hind, F_3,13_ = 11.66, *p* < 0.001; right front, F_3,13_ = 60.9, p < 0.001; right hind, F_3,13_ = 14.27, *p* < 0.001. **B** Latency to fall off the rotarod was analyzed at two time points, prior to injection (35 days) of age and 40 days post injection (80 days of age). 35 days of age: *Gba*^*+/+*^*;Gba*^*tg*^ + PBS (*n* = 10), *Gba*^*−/−*^*;Gba*^*tg*^ + PBS (*n* = 10), *Gba*^*−/−*^*;Gba*^*tg*^ + AAV-dGCase (*n* = 10) and *Gba*^*−/−*^*;Gba*^*tg*^ + AAV*-*wtGCase (*n* = 8). Mixed ANOVA test was applied to determine the effect of treatment on the beam versus time and the combined effect of time and treatment. Treatment; F_3,61_ = 3.00, *p* < 0.05, timepoint; F_1,61_ = 16.77, *p* < 0.001; treatment:timepoint, F_3,61_ = 5.15, *p* < 0.01. Tukey HSD post-hoc test showed no difference in time-to-fall-off for the first timepoint, whereas there was a significant difference between the groups at the second timepoint. The test also revealed differences between *Gba*^*−/−*^*;Gba*^*tg*^ + AAV-dGCase *versus Gba*^*−/−*^*;Gba*^*tg*^ + PBS, regardless of time (*p* < 0.05*)*^.^ Boxes represent lower quartile, median and upper quartile (*black*). The whiskers represent the minimum and maximum values, up to 1.5-times the interquartile range from the bottom or the top of the box to the furthest data point within that distance, thus excluding outliers. The mean is in *red*. *p ≤ 0.05; **p ≤ 0.01; ***p ≤ 0.001.

At 80 days of age, motor function was analyzed using two balance and coordination tests, the rotarod and the beam walk tests (Fig. 3). These motor tests generated two parameters, namely percent foot slips for each foot (beam walk, Fig. 3A), and latency to fall-off (rotarod, Fig. 3B), with poor motor function indicated by a higher percentage of foot slips in the beam walk test and a shorter latency to fall off in the rotarod test. *Gba*^*−/−*^*;Gba*^*tg*^ mice injected with either PBS or with AAV-wtGCase slipped off the beam significantly more than *Gba*^*+/+*^*;Gba*^*tg*^ injected with PBS, whereas the percentage of foot slips of *Gba*^*−/−*^*;Gba*^*tg*^ mice injected with AAV-dGCase was reduced almost to control levels (Fig. 3A). Latency to fall-off was compared in mice at 35 *versus* 80 days of age (Fig. 1A). A significant shorter latency to fall off the rod was evident at 80 days of age compared to 35 days of age for *Gba*^*−/−*^*;Gba*^*tg*^ mice injected with PBS and *Gba*^*−/−*^*;Gba*^*tg*^ injected with AAV-wtGCase (Fig. 3B), indicating a progressive worsening of disease symptoms. In contrast, there was no difference between 35 and 80 day-old mice for *Gba*^*+/+*^*;Gba*^*tg*^ mice injected with PBS and for *Gba*^*−/−*^*;Gba*^*tg*^ mice injected with AAV-dGCase (Fig. 3B). A movie documenting mouse motor function on the beam walk test can be found in Supplementary Material along with all data points (Supplementary Table 3)

**Fig. 4:**
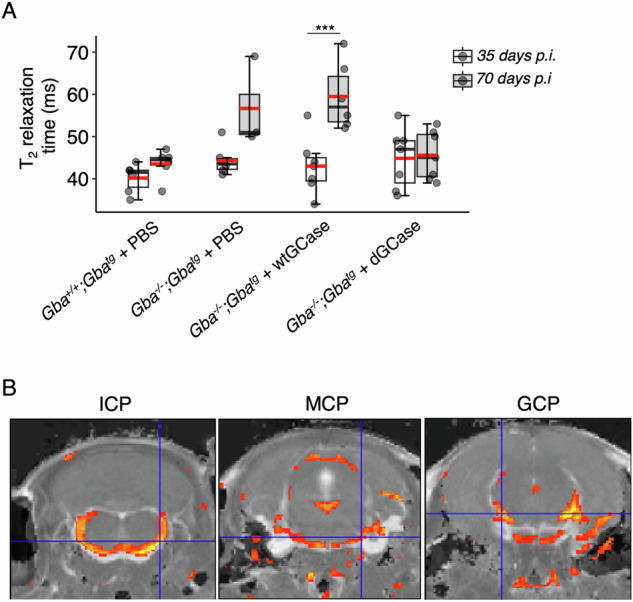
MRI analysis. **A** Quantification of MRI T_2_ maps 35- and 70-days post-injection (p.i.). *Gba*^*+/+*^*;Gba*^*tg*^ + PBS, *n* = 6 for 35 days p.i. and *n* = 7 for 70 days p.i.; *Gba*^*−/−*^*;Gba*^*tg*^ + PBS, *n* = 6 for 35 days p.i. and *n* = 3 70 days p.i; *Gba*^*−/−*^*;Gba*^*tg*^ + AAV-dGCase, *n* = 7 for both 35 and 70 p.i.; *Gba*^*−/−*^*;Gba*^*tg*^ + AAV-dGCase, *n* = 7 for both 35 and 70 p.i. Mixed ANOVA test was applied to determine the effect of treatment on T_2_ relaxation time in the VPM/VPN versus time and the combined effect of time and treatment. Treatment; F_3,40_ = 4.84, *p* < 0.01, timepoint; F_1,40_ = 18.80, *p* < 0.001 and treatment:timepoint; F_3,40_ = 4.68, *p* < 0.01. Tukey HSD post-hoc test showed a significant difference in T_2_ relaxation time in the VPM/VPN at 70 days p.i. between *Gba*^*−/−*^*;Gba*^*tg*^ + AAV-wtGCase *versus Gba*^*+/+*^*;Gba*^*tg*^ + PBS (*p* = 0.001) and to *Gba*^*−/−*^*;Gba*^*tg*^ + AAV-dGCase (*p* < 0.01). Boxes represent lower quartile, median and upper quartile (*black*). The whiskers represent the minimum and maximum values, up to 1.5-times the interquartile range from the bottom or the top of the box to the furthest data point within that distance, thus excluding outliers. The mean is in *red*. *p ≤ 0.05; **p ≤ 0.01; ***p ≤ 0.001. **B** Three representative coronal slices of T_2_ maps. The yellow-orange areas represent the most prominent areas characterized by a significant reduction (*p* < 0.05) in T_2_ relaxation time after injection of dGCase. The analysis was performed in unbiased manner. Brain areas: ICP, inferior cerebellar peduncle; MCP, middle cerebellar peduncle; GCP, general cerebellar peduncle.

Mice were next examined by MRI. A trend towards decreased cerebellar volume in *Gba*^*−/−*^*;Gba*^*tg*^ mice injected with PBS *versus Gba*^*−/−*^*;Gba*^*tg*^ mice injected with AAV-dGCase was observed (Supplementary Table 3), although the variability in the cerebellar volume between the groups rendered it difficult to reach a definitive conclusion regarding the effect of AAV-dGCase on cerebellar pathology. In contrast, unambigious differences were seen with respect to the T_2_ relaxation time, which can be used as a surrogate marker for (neuro-)inflammation [22], in the ventral posterior nucleus [ventral posteromedial/posterolateral (VPM/VPL)], an area prevously shown to display sigificant neuroinflammation in GD [11, 23]. Thus, the T_2_ relaxation time increased somewhat between 40- and 70-days post injection in *Gba*^*−/−*^*;Gba*^*tg*^ mice injected with either PBS or with AAV-wtGCase, but not in *Gba*^*+/+*^*;Gba*^*tg*^ mice injected with PBS or in *Gba*^*−/−*^*;Gba*^*tg*^ mice injected with AAV-dGCase (Fig. 4A). The white matter was particularly affected, mainly in the inferior cerebellar peduncle (ICP), middle cerebellar peduncle (MCP) and general cerebellar peduncle (GCP) (Fig. 4B).

**Fig. 5:**
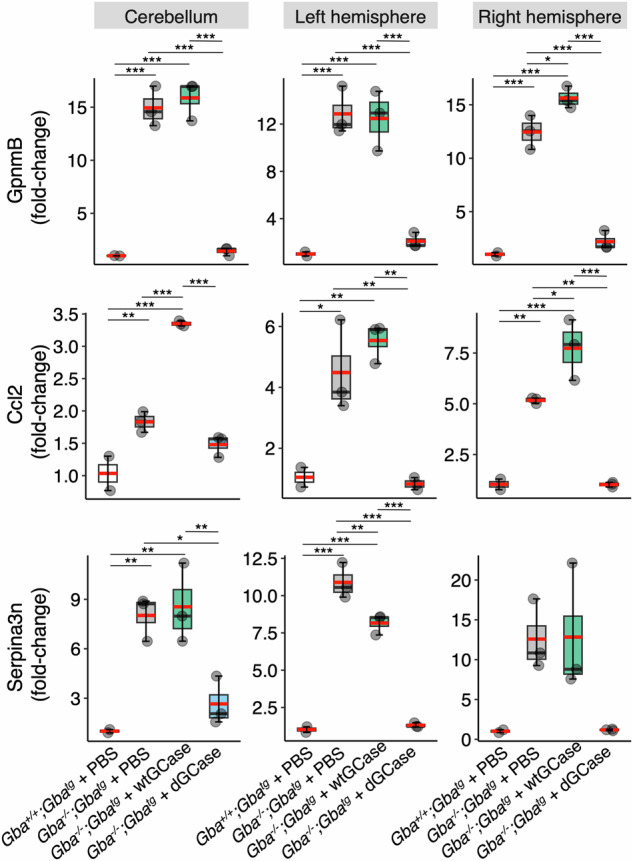
Real-time PCR analysis of inflammatory genes. *Gba*^*+/+*^*;Gba*^*tg*^ + PBS, *n* = 2, 171 and 261 days of age; *Gba*^*−/−*^*;Gba*^*tg*^ + PBS, *n* = 3, 105, 110 and 115 days of age; *Gba*^*−/−*^*;Gba*^*tg*^ + AAV-wtGCase, *n* = 3, 103, 112 and 140 days of age; *Gba*^*−/−*^*;Gba*^*tg*^ + AAV-dGCase, *n* = 3, 203, 221, 221 days of age. Boxes represent lower quartile, median and upper quartile (*black*). The whiskers represent the minimum and maximum values, up to 1.5-times the interquartile range from the bottom or the top of the box to the furthest data point within that distance, thus excluding outliers. The mean is in *red*. *p ≤ 0.05; **p ≤ 0.01; ***p ≤ 0.001.

Finally, real-time PCR analysis revealed a large reduction in levels of three genes, *GpnmB*, *Ccl2* and *Serpina3n*, previously shown to be elevated in neurological mouse models of GD [24–26]. Unlike GlcCer levels and GCase levels, the expression of these 3 genes was reduced in all three brain regions (cerebellum, left and right hemispheres) in *Gba*^*−/−*^*;Gba*^*tg*^ mice injected with AAV-dGCase compared to *Gba*^*−/−*^*;Gba*^*tg*^ mice injected with PBS or with AAV-wtGCase (Fig. 5).

## Discussion

The current study focuses on the potential use of a computationally-enhanced version of a human lysosomal enzyme for use in gene therapy. Due to concerns about the immunogenicity of proteins with altered amino acid sequences, this approach has not been used previously. However, in the case of a protein expressed uniquely in the brain, an adverse immunological response is far less likely, and moreover, AAVrh10 generally display low immunogenicity [27].

While our data are very promising, in particular the significant extension of mouse life-span, there are nevertheless a number of issues that need to be addressed. Perhaps the most confounding observation is the lack of an effect of the wtGCase on the lifespan of *Gba*^*−/−*^*;Gba*^*tg*^ mice. Only two previous studies have attempted a gene therapy approach in mice that model neurological forms of GD [7, 28] using an acute type 2 mouse, which normally only lives for 15 days [29]. In one of the studies, upon fetal intracranial injection of an AAV vector encoding wtGCase, mice lived for at least 18 weeks. The reason for the lack of efficacy of wtGCase in *Gba*^*−/−*^*;Gba*^*tg*^ mice is unclear. We speculate that it may be related to the time of the injection (36–38 days after birth) or a lower rate of diffusion from the site of injection. In our view, the enhanced thermostability and expressibility of dGCase [8] and its enhanced ability to clear GlcCer relative to wtGCase [8], suggest that its greater in vivo efficacy compared to wtGCase may be due to its enhanced stability. Unfortunately, we were unable to examine the spread of the enzyme from the site of injection due to the lower immunonogenicity towards dGCase of a commercial antibody generated against wtGCase.

Some other inconsistencies are apparent from our data. For instance, while the viral vector was injected into the left hemisphere, a slightly better response was noted in the right hemisphere, at least for reduction of GlcCer levels and elevation of GCase activity, although three pathogenic markers were reduced similarly in all three brain regions. However, the biochemical changes detected in the right hemisphere were evidently sufficient to increase mouse life span, revert motor function to levels similar to those detected in *Gba*^*+/+*^*;Gba*^*tg*^ mice injected with PBS, and reduce inflammation, as detected by MRI. Thus, while the efficacy of dGCase in reducing GD symptoms in *Gba*^*−/−*^*;Gba*^*tg*^ mice is unquestionable, further analysis and testing are required. It should be noted that intracerebroventricular injection has been shown to lead to uneven distribution of the vector [27].

Finally, we note that the prospect of using computationally enhanced enzymes in other LSDs or more broadly in other monogenetic diseases, is extremely attractive. Such enzymes are likely to be more stable in the brain and thus remain longer after a one-off injection. An enzyme and a vaccine immunogen stabilized using the same computational strategy are now being tested in animal studies and clinical trials. Our study may open the door for similarly stabilized forms of enzymes to be used to treat rare monogenetic diseases.

## Supplementary information

Supplementary material.

SUPPLEMENTAL TABLE 3

Supplementary movie

## Data Availability

Data used in this study are available within the article and its Supplementary material.
