# Supplementary material for: Efficacy of an AAV vector encoding a thermostable form of glucocerebrosidase in alleviating symptoms in a Gaucher disease mouse model
Source: Gene Ther. 2024 Aug 15;31(9-10):439–44. doi: 10.1038/s41434-024-00476-8 (PMC11399100; doi:10.1038/s41434-024-00476-8)
Supplement: Supplementary file 1 — Supplementary material. [file 41434_2024_476_MOESM1_ESM.docx]

**Efficacy of an AAV vector encoding a thermostable form of glucocerebrosidase in alleviating symptoms in a Gaucher disease mouse model**

Ivan Milenkovic^1,2,#^, Shani Blumenreich^1,#^, Ariel Hochfelder^1^, Aviya Azulay^1^, Inbal E. Biton^3^, Mirie Zerbib^3^, Roni Oren^3^, Michael Tsoory^3^, Tammar Joseph^1^, Sarel J. Fleishman^1^ and Anthony H. Futerman^1,*^

**Supplementary Material**

**Supplementary Table 1. Primers used for genotyping *Gba^+/+^;Gba^tg^* and *Gba^-/-^;Gba^tg^* mice**.

| Gene | Primers |
| --- | --- |
| *Gba* | Common R’-ACACGACCACAACTGCAGAG  WT F‘-TCCTCACCTCCTCAGATGCT  Null F’-CTCGTGCTTTACGGTATCGC |
| Tg | Common F’-CTCGTGATCTGCAACTCCA  WT R’- ATGGATATGAAGTACTGGGCTCT  Tg R’-CTAATGCGCTGTTAATCAC |

**Supplementary Table 2. Primers used for Real-time PCR.**

| Gene | Primers |
| --- | --- |
| *GpnmB* | F’-AGGAACACGGTCAAGGGCAA  R’-CTGGTCAGAGGGAAGGCCAA |
| *Ccl2* | F’-TCACCTGCTGCTACTCATTCACCA  R’-AGCACAGACCTCTCTCTTGAGCTT |
| *Serpina3n* | F’-TTGCCCCCTTTATAGCCAAGA  R’-AGACCCACAGACAGGCTCAA |

**Supplementary Table 3. Data documenting individual mice.** See excel file.

**Supplementary movie showing motor function on the beam test.** A representative video of mice from each group walking over a plank during the beam walk test.
